# Spreading brain lesions in a familial Creutzfeldt-Jakob disease with V180I mutation over 4 years

**DOI:** 10.1186/1471-2377-12-144

**Published:** 2012-11-24

**Authors:** Kentaro Deguchi, Motonori Takamiya, Shoko Deguchi, Nobutoshi Morimoto, Tomoko Kurata, Yoshio Ikeda, Koji Abe

**Affiliations:** 1 Department of Neurology, Graduate School of Medicine, Dentistry and Pharmaceutical Sciences, Okayama University, 2-5-1 Shikata-cho, kitaku, Okayama 700-8558, Japan

**Keywords:** Familial Creutzfeldt-Jakob disease, V180I, Magnetic resonance imaging, Electroencephalogram

## Abstract

**Background:**

We report a female patient with familial Creutzfeldt-Jakob disease with V180I mutation (fCJD with V180I), who was serially followed up with magnetic resonance imaging (MRI) and electroencephalogram (EEG) for up to four years.

**Case presentation:**

At 6 months after the onset, diffusion-weighted images (DWI) and fluid-attenuated inversion recovery (FLAIR) of brain MRI revealed an increased signal intensity in the bilateral frontal, temporal, and parietal cerebral cortex with left dominancy except for the occipital lobe. However, her follow-up MRI at four years showed the high-signal regions spreading to the occipital cerebral cortex in DWI and FLAIR images, and bilateral frontal cerebral white matter in FLAIR images. EEG showed a progressive and general slow high-voltage rhythm from 7–8 to 3–5 c/s over four years, without evidence of periodic synchronous discharge. These findings correspond to the symptom progression even after akinetic mutism at 18 months.

**Conclusion:**

We suggest that serial MRI and EEG examinations are useful for early diagnosis of fCJD with V180I and for monitoring disease progression.

## Background

Creutzfeldt-Jakob disease (CJD) is a transmissible neurodegenerative disease caused by abnormal prion protein (PrP). Magnetic resonance imaging (MRI) in patients with CJD have shown hyperintense signal abnormalities in the striatum or thalamus on T2-weighted images [1]. In addition, diffusion-weighted imaging (DWI) has been reported to be a useful technique for early diagnosis of CJD [2-5]. Recently, Geshcwind et al. reported that the pattern of fluid-attenuated inversion recovery (FLAIR)/DWI hyperintensity and restricted diffusion can differentiate sporadic CJD from other non-prion causes of rapidly progressive dementia with a high sensitivity and specificity [6]. Approximately 10–15% of all CJD cases are familial CJD (fCJD), with variable clinical features and MRI findings [7]. fCJD with a causative point mutation of valine to isoleucine at codon 180 (V180I) in the PrP gene shows a unique MRI finding of marked high-intensity areas with ribboning in the cerebral cortex at an early stage, except for the medial occipital and cerebellar cortices [7]. However, serial changes on MRI and electroencephalogram (EEG) during the long follow-up period of CJD have not been fully reported. Herein, we report serial changes of MRI and EEG findings in relation to the clinical signs and symptoms in a case of fCJD with a causative point mutation of V180I in the PrP gene (fCJD with V180I) over a four year follow up.

## Case presentation

A 79 year old Japanese woman first visited our Okayama University Hospital in 2007 for the evaluation of cognitive decline and walking unsteadiness that gradually progressed over six months. She had no past medical history requiring blood transfusion or receiving other human dura mater. She had not traveled abroad. None of her family suffered from similar symptoms or history.

Physical examination of the patient was normal. Psychological examination revealed lack of initiative, severe disorientation, acalculia, and short-term memory loss. Mini-mental state examination (MMSE) was 12 out of 30, mainly involving orientation, recent memory, and attention. Neurological examination of this patient showed no abnormal findings in cranial nerves and the autonomic system. However, bradykinesia was noted in her body, face, and quadro extremities. Muscle tonus was increased with rigidity in her four limbs and neck. Increased deep tendon reflexes were observed in the bilateral upper limbs without pathological reflexes. Assessment of the sensory system was difficult owing to dementia. She was able to stand with some support, but was unable to walk. Involuntary movement such as myoclonus or startle reaction was not observed.

**Figure 1:**
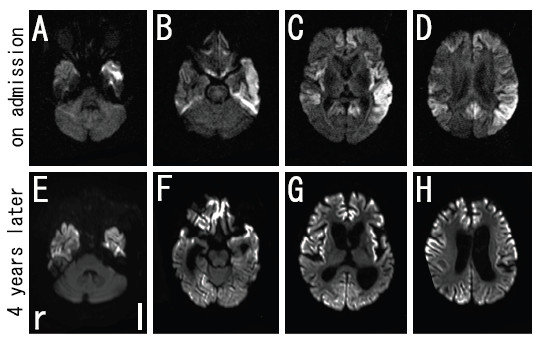
**DWI images of brain MRI in the present case. **Increased signal intensity in the bilateral frontal, temporal, and parietal cerebral cortex with left dominancy, except for occipital lobe, was observed on admission with axial imaging (**A**-**D**). Note that the high-signal region spread to the occipital cerebral cortex at four years after the onset (**E**-**H**).

**Figure 2:**
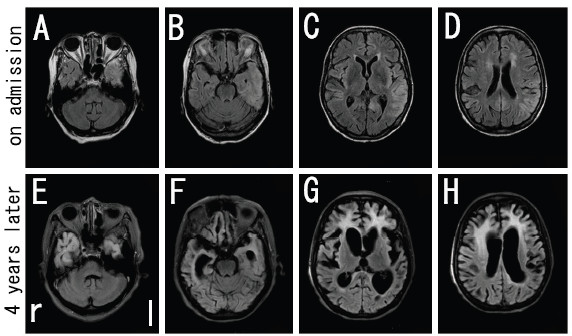
**FLAIR images of brain MRI in the present case. **Increased signal intensity was observed on admission (**A**-**D**). Note that the high-signal region spread to the occipital cerebral cortex and the frontal white matter at four years after the onset with a marked ventricular dilatation (**E**-**H**).

**Figure 3:**
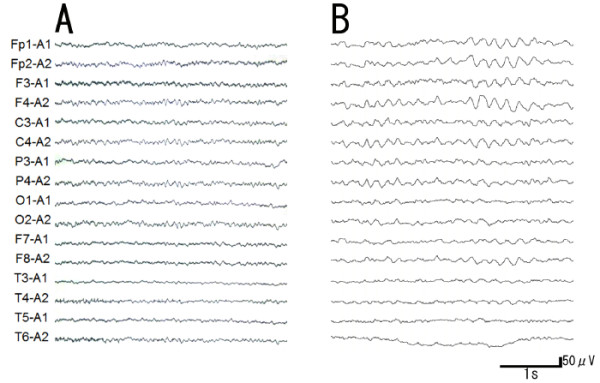
**Electroencephalogram (EEG) in the present case. **Note that EEG changed from a generalized slow basic rhythm at 7–8 c/s on admission (**A**) to a general slower high-voltage basic rhythm at 3–5 c/s after four years, but without PSD (**B**).

Biochemical tests of blood showed no abnormal findings. Cerebrospinal fluid (CSF) analysis revealed an elevated neuron-specific enolase (NSE) to 74.1 ng/ml (normal <35.0 ng/ml), although CSF cell count and protein and serum NSE (15.9 ng/ml, normal <16.3 ng/ml) were normal. DWI (Figure 1A-D) and fluid-attenuated inversion recovery (FLAIR) (Figure 2A-D) of brain MRI revealed increased signal intensity in the bilateral frontal, temporal, and parietal cerebral cortex with left dominancy, but no change in the occipital lobe. EEG showed a generalized slow basic rhythm at 7–8 c/s without periodic synchronous discharge (PSD; Figure 3A). Prion protein gene analysis of peripheral blood was then performed with informed consent, which revealed a G to A transition at codon 180 (GTC: Val → ATC: Ile; V180I). Her CSF on admission was positive for 14-3-3 protein and total tau protein (>1300 pg/ml).

Intellectual performance of the patient deteriorated progressively, and she developed akinetic mutism with stronger rigidity of four limbs at 18 months after onset. Follow-up MRI at 54 months after onset showed spread of the high-signal region to the occipital cerebral cortex in DWI (Figure 1E-H) and FLAIR images (Figure 2E-H), and evidence of bilateral frontal cerebral white matter lesions in FLAIR images (Figure 2E-H). EEG at that time showed a general slower high-voltage basic rhythm than 4 years prior (to 3–5 c/s), but still without PSD (Figure 3B). Although the patient remained in a vegetative state in a chronic care unit at 60 months after the onset, she still maintained oral intake with assistance.

## Conclusions

We report a patient of familial Creutzfeldt-Jakob disease (fCJD) with a causative point mutation of V180I in the prion protein gene (fCJD with V180I). A high-signal region on brain MRI was located at the cerebral cortex in the early stage (Figure 1A-D; Figure 2A-D), which progressively spread to the occipical lobe over the parieto-occipical sulcus and finally to the bilateral frontal white matter over four years follow-up (Figure 1E-H; Figure 2E-H), but without PSD of EEG. This V180I mutation is the most common cause of fCJD in Japan, with only two cases reported from Europe [8,9]. Compared with sporadic CJD (sCJD), fCJD with V180I showed (1) older onset age, (2) slower disease progression, (3) unique clinical symptoms such as frequent cognitive dysfunction without visual or cerebellar symptoms, (4) a lower positive rate of NSE and 14-3-3 protein in CSF, and (5) a lack of PSD in EEG throughout the course of the disease [7]. Our patient was compatible with the clinical and laboratory characteristics of this fCJD with V180I.

DWI is a useful technique for the early diagnosis of CJD [2-5], and diffuse cortical high-intensity signals in DWI and FLAIR image are characteristic feature of fCJD with V180I. However, serial changes on DWI and FLAIR image during the long-term period of fCJD with V180I have not been fully reported. Kono et al. reported that the abnormal lesions of a fCJD patient with V180I began from the left cerebral cortex to the contralateral cerebral cortex and the basal ganglia, with sparing the occipital, parieto-occipital lobes, and the cerebellum until the terminal stage at 25 months after the onset [10]. However, the present case involved involved the occipital cerebral cortex and the frontal white matter over 4 years follow-up.

Serial EEG of fCJD with V180I usually shows a diffuse slow basic pattern without PSD, although most patients of sCJD demonstrate PSD in EEG, which is an accepted diagnostic marker for sCJD [7]. The present case showed a similar course of EEG to typical fCJD with V180I. The general slower high-voltage basic rhythm of EEG (Figure 3B) at four years after the onset may reflect a severe deterioration in brain function such as akinetic mutism, not but apallic syndrome.

Spongiform degeneration is a typical pathologic change of CJD characterized by vacuoles in the neuropil, gliosis, and nerve cell loss in the gray matter [11,12]. In fCJD cases with V180I, increased DWI signals and a hypoperfusion region on single photon emission computed tomography (SPECT) could reflect such spongiform changes [11]. In addition, Iwasaki et al. reported a widespread fibrous gliosis with hypertrophic astrocytosis and proliferation of macrophages in the cerebral white matter in the brain of fCJD with V180I [13]. Thus, in the present case of fCJD with V180I, the prominent white matter lesions of the frontal lobe might reflect secondary axonal degeneration due to widespread cerebral neocortical involvement (Figure 2G,H).

In conclusion, during the long follow-up period of fCJD with the present V180I case, MRI finding spread from the frontal, temporal, and parietal cortical lobe to occipital cortical lobe, with the possibly secondary change of the frontal white matter. In addition, EEG findings corresponded the symptom progression even after akinetic mutism at 18 months. Thus, the present case suggests that serial DWI, FLAIR, and EEG follow-up is useful for early diagnosis of CJD and for monitoring disease progress.

## Consent

Written informed consent was obtained from the family of the patient for publication of this case report and accompanying images. A copy of the written consent is available for review by the Editor-in-Chief of this journal.

## Abbreviations

CJD: Creutzfeldt-Jakob disease; fCJD with V180I: fCJD with a causative point mutation of V180I in the prion protein gene; CSF: Cerebrospinal fluid; DWI: Diffusion-weighted images; EEG: Electroencephalogram; fCJD: Familial CJD; FLAIR: Fluid-attenuated inversion recovery; MMSE: Mini-mental state examination; MRI: Magnetic resonance imaging; NSE: Neuron-specific enolase; PrP: Prion protein; PSD: Periodic synchronous discharge; sCJD: Sporadic CJD; V180I: G to A transition at codon 180.

## Competing interests

All authors declare that they have no competing interests.

## Authors contributions

KD examined and evaluated the patient, and drafted the manuscript. MT, SD, NM, TK, YI, and KA made contributions to treat the patient. KA participated in the design of the case report and helped to draft the manuscript. All authors read and approved the final manuscript.

## Pre-publication history

The pre-publication history for this paper can be accessed here:

http://www.biomedcentral.com/1471-2377/12/144/prepub
